# Supplementary material for: Clinical Characteristics and Risk Factors of Tuberculosis in Children and Adolescents in Xinjiang, China: A Retrospective Analysis
Source: Trop Med Infect Dis. 2025 Oct 16;10(10):293. doi: 10.3390/tropicalmed10100293 (PMC12568225; doi:10.3390/tropicalmed10100293)
Supplement: Supplementary file 1 [file tropicalmed-10-00293-s001.zip › tropicalmed-3889459-supplementary.pdf]

Table S1

Clinical Data Collection Form

Characteristics	Options
Date of admission	
Gender	Male ( ) Female ( )
Age, y	
Residence	City ( ) County ( ) Rural ( )
BCG vaccination	Yes ( ) No ( ) Unknown( )
Time from onset of symptoms to hospital visit, d	
TB type	PTB ( ) EPTB：Tuberculous meningitis ( ) Lymphatic TB ( ) Osteoarticular TB ( ) Abdominal TB ( ) Intestinal TB ( ) Others ( )
Contact history	Yes ( ) No ( ) Unknown ( )
Severity of TB	Severe ( ) Non-severe ( )
Clinical manifestations	Fever ( ) Cough ( ) Sputum production ( ) Fatigue ( ) Loss of appetite ( ) No weight gain or loss ( ) Night sweats ( )
Laboratory Tests	TST ( ) IGRA ( ) smear microscopy ( ) Culture ( ) molecular detection ( )
